# Delivered dose can be a better predictor of rectal toxicity than planned dose in prostate radiotherapy

**DOI:** 10.1016/j.radonc.2017.04.008

**Published:** 2017-06

**Authors:** L.E.A. Shelley, J.E. Scaife, M. Romanchikova, K. Harrison, J.R. Forman, A.M. Bates, D.J. Noble, R. Jena, M.A. Parker, M.P.F. Sutcliffe, S.J. Thomas, N.G. Burnet

**Affiliations:** a Cambridge University Hospitals NHS Foundation Trust, Department of Oncology, United Kingdom; b Department of Medical Physics and Clinical Engineering, Cambridge University Hospitals NHS Foundation Trust, United Kingdom; c Department of Engineering, University of Cambridge, United Kingdom; d Department of Oncology, University of Cambridge, United Kingdom; e Cambridge Clinical Trials Unit, Cambridge University Hospitals NHS Foundation Trust, United Kingdom; f Cavendish Laboratory, University of Cambridge, United Kingdom

**Keywords:** Rectal toxicity, VoxTox, Dose–surface maps, Delivered dose, Prostate radiotherapy

## Abstract

**Background and purpose:**

For the first time, delivered dose to the rectum has been calculated and accumulated throughout the course of prostate radiotherapy using megavoltage computed tomography (MVCT) image guidance scans. Dosimetric parameters were linked with toxicity to test the hypothesis that delivered dose is a stronger predictor of toxicity than planned dose.

**Material and methods:**

Dose–surface maps (DSMs) of the rectal wall were automatically generated from daily MVCT scans for 109 patients within the VoxTox research programme. Accumulated-DSMs, representing total delivered dose, and planned-DSMs, from planning CT data, were parametrised using Equivalent Uniform Dose (EUD) and ‘DSM dose-width’, the lateral dimension of an ellipse fitted to a discrete isodose cluster. Associations with 6 toxicity endpoints were assessed using receiver operator characteristic curve analysis.

**Results:**

For rectal bleeding, the area under the curve (AUC) was greater for accumulated dose than planned dose for DSM dose-widths up to 70 Gy. Accumulated 65 Gy DSM dose-width produced the strongest spatial correlation (AUC 0.664), while accumulated EUD generated the largest AUC overall (0.682). For proctitis, accumulated EUD was the only reportable predictor (AUC 0.673). Accumulated EUD was systematically lower than planned EUD.

**Conclusions:**

Dosimetric parameters extracted from accumulated DSMs have demonstrated stronger correlations with rectal bleeding and proctitis, than planned DSMs.

In prostate radiotherapy, the correlation between dose to rectum and toxicity has been the focus of many research studies [1], [2], [3], [4], [5], [6], [7]. The rectum is one of the dose-limiting organs when planning intensity-modulated radiotherapy (IMRT) to the prostate due to the risk of radiation-induced adverse effects. Modern systems for inverse IMRT treatment planning iteratively seek to achieve an optimal plan, delivering maximal dose to the tumour volume and minimal dose to healthy organs. Current normal tissue complication probability (NTCP) models and conventional treatment planning constraints are based upon dose–volume histogram (DVH) data to minimise the risk of toxicity. With ever improving disease control [8], [9] and survival rates [10], post-treatment quality of life becomes an increasingly significant consideration during treatment planning, alongside target coverage.

The DVH-based approach to radiotherapy treatment planning has been criticised for lacking in spatial dose consideration [2]. Consequently, accumulation of DVHs is not dosimetrically representative and results in false overestimations of dose. A review by Landoni et al. [11] emphasises the need to assess associations between spatial dose patterns and late toxicity [12], particularly as results may reveal inhomogeneous intra-organ radiosensitivities.

Several groups have explored alternative approaches for parametrisation of dose distributions in order to establish links with toxicity. Methods have included dose–surface histograms [1], [5], [13], [14], dose–surface maps [1], [5], [15], dose–line histograms [14], principal component-based pattern analysis [16], and voxel-based approaches for identifying rectal subregions [2], [6], [7]. These studies have been limited in their analysis by the availability of planned dose data only, based on a single anatomical snapshot in time.

A common recommendation in the literature has been the need to establish dose-toxicity models based on delivered dose [17]. However, this has proven technically challenging to date due to hardware and software limitations. These challenges have been addressed within the VoxTox Research Programme [18] where contours generated from on-treatment megavoltage computed tomography (MVCT) image guidance scans are used to calculate daily delivered dose. This approach has made it possible to account for the effect of interfractional anatomical variation. Total delivered dose can be estimated by accumulating daily delivered dose throughout the course of radiotherapy. Studies by the VoxTox group have demonstrated that the rectum moves more than previously predicted based on estimates from prostate motion [19], and that planned dose is not equal to delivered dose [20].

The dose–surface map (DSM) approach has been implemented within this study as a solution enabling meaningful accumulation and conservation of geometric information, an advantage over the DVH methodology. The concept of accumulating DSMs to estimate total delivered dose has been applied previously for the bladder [13]. By extracting spatial parameters from DSMs of delivered dose, and linking with the archive of patient follow-up data available within VoxTox, it was hypothesised that stronger correlations could be established with late toxicity than previously achievable using planned dose alone. Ultimately, improved dose-toxicity modelling based on delivered dose could facilitate real-time in silico prediction of NTCP within the clinical pathway.

## Material and methods

### VoxTox study design & patient information

The VoxTox research programme is an observational study linking radiation dose to toxicity outcomes [18], [20]. It received approval from the National Research Ethics Service (NRES) Committee East of England (13/EE/0008) in February 2013 and is part of the UK Clinical Research Network Study Portfolio (UK CRN ID 13716).

One hundred and nine prostate cancer patients were selected from the discovery cohort of the VoxTox research programme [18]. This cohort (Table 1) comprised patients treated prior to the formal collection of baseline data, but for whom prospective follow-up data of at least 2 years were available (median 4 years). Early VoxTox patients were selected based on expected benefit from IMRT rather than conventional 3D conformal radiotherapy. Patients in this study were included on the basis of availability of pre-existing toxicity status from clinical notes, or no reported toxicity, and was limited to those prescribed IMRT to a dose of 74 Gy in 37 fractions, the standard of care in the UK at the time [21]. VoxTox patients are treated with TomoTherapy® (Accuray, Sunnyvale, CA). Manual contouring of the anatomy on the kilovoltage computed tomography (kVCT) planning scan was performed according to local procedures [19], adapted from clinical trials. Daily MVCT image guidance scans were acquired immediately prior to treatment delivery for the purposes of online target localisation [22]. Following our department protocol, scans were inspected for rectal dilation and if deemed excessive, remedial action was taken prior to delivery of radiation therapy [23].

**Table 1:** Baseline characteristics for the 109 VoxTox participants. Prescribed dose to the prostate was 74 Gy in 37 fractions over 7.5 weeks. All patients were treated with androgen deprivation therapy. IBD = inflammatory bowel disease, IQR = interquartile range, PSA = prostate-specific antigen, SD = standard deviation.

Clinical data for VoxTox patients (*n* = 109)	
**Age, years**	
Median (IQR)	68 (64–71)
Range	51–80

**T stage,***n***(%)**	
T1A/T1B/T1C/T1X	24 (22%)
T2A/T2B/T2C/T2X	34 (31%)
T3A/T3B/T3X	45 (41%)
T4	0
Not known	6 (6%)

**Gleason score,***n***(%)**	
⩽6	23 (21%)
7	44 (40%)
⩾8	39 (36%)
Not known	3 (3%)

**PSA (ng/ml)**	
Median (IQR)	11 (7–20)
Mean (SD)	20 (30)
Not known	3 (3%)

**Clinical history**	
Diabetes	10 (9%)
Hypertension	35 (32%)
IBD or diverticular disease	7 (6%)
Previous pelvic surgery	7 (6%)
Haemorrhoids past 12 months	3 (3%)
Any previous TURP	9 (8%)
Not known	9 (8%)

### Dose–surface map construction & dose accumulation

Within the VoxTox research programme, MVCT scans are multifunctional; primarily for the purpose of routine image guidance, they also provide a platform for calculation of delivered dose. The rectum was identified on each MVCT image series using an in-house autocontouring system based on a customised Chan-Vese segmentation algorithm [24]. Delivered dose was independently calculated using a locally implemented ray-tracing algorithm [25], [26] and the rectal contour-of-the-day, accounting for inter-fraction motion. Automation and integration of dose calculation and contouring systems were essential for large-scale processing of the 4142 scans in this study.

Planned and daily DSMs were generated based on algorithms described by Buettner et al. [1] and Murray et al. [15]. The rectal wall was considered the structure of interest, and was treated as a tubular surface rather than a volume. Contours were virtually ‘cut’ along the superior–inferior axis and ‘unfolded’ to a two-dimensional plane. The ‘cutting point’ was identified as the point on the contour surface directly posterior to the centre of mass of the rectal outline, on each CT slice [20].

**Fig. 1:**
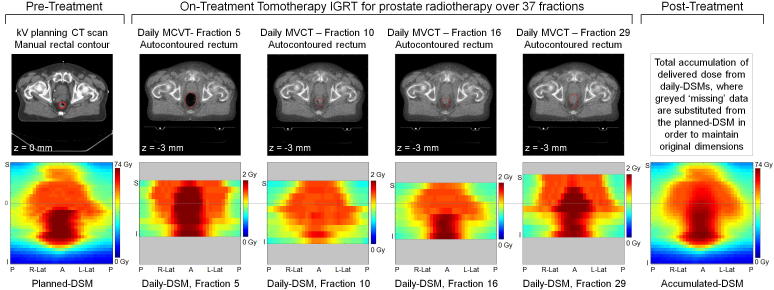
Generation of planned, daily and accumulated dose surface maps.

The height of the planned-DSM was defined by the number of slices of the manually contoured rectum on the kV planning scan (slice thickness 3 mm). The circumference of the rectal contour on each slice was normalised such that the unfolded width of the planned-DSM was equal to the height. Daily delivered DSMs calculated from the image-guidance MVCT scans (slice thickness 6 mm) were normalised to the same width as the planned-DSM but were restricted in height by the field of view (FOV), resulting in a shorter DSM, as shown in Fig. 1.

Rectal DSMs were calculated for each treatment fraction, and corrected for daily couch shifts. For the purposes of dose accumulation, any ‘missing’ dose data cropped by the restricted FOV superiorly or inferiorly were substituted from the planned-DSM [20] in order to maintain common dimensions between final accumulated-DSM and planned-DSM. The final accumulated-DSM was resampled to match the 3 mm resolution of the planned-DSM, producing an easily comparable and interpretable spatial representation of total delivered dose to the rectal wall throughout the course of prostate radiotherapy [25] (illustrated in Fig. 1).

The use of planned-DSM data as a surrogate beyond the boundaries of the MVCT FOV was considered an acceptable estimate under the assumption that the relative anatomical motion of the rectum becomes more confined by surrounding musculature as the distance from the prostate increases [20]. However, this could have reduced potential differences between planned and accumulated dose, and was a limitation of the analysis.

### Dose parameters & clinical endpoints

Dose was parametrised from DSMs using two methods implemented in MATLAB® (MathWorks®, Natick, MA):

1. Calculation of Equivalent Uniform Dose (EUD)
2. Fitting of DSM ‘dose-widths’ to discrete isodose clusters

EUD reduces the dose information extracted from the DSMs to a single generalised value which allows comparison between inhomogeneous dose distributions [27]. An ‘a’ value of 11.11 was used in the EUD calculation [28]. Spatial dose information was generated by reproducing Buettner’s ellipse-fitting method [1], reporting the most significant dose quantifier, the lateral extent, termed here the ‘DSM dose-width’.

For a given isodose level, a binary image was created from the DSM by assigning a pixel value of 1 to doses greater than or equal to the nominated isodose, with lower doses assigned a value of 0. An ellipse was then fitted to the largest central cluster. The maximum lateral extent of the ellipse was projected onto the DSM axis, accounting for any rotation with respect to the DSM coordinate system. The resulting DSM dose-width, expressed as a the percentage of total normalised DSM width, allowed parametrisation of the geometrical dose distribution which would have been masked using a DVH approach.

For each patient, EUD and DSM dose-widths for isodose levels of 30, 40, 50, 60, 65, and 70 Gy were calculated from planned-DSM and accumulated-DSM. Doses less than 30 Gy were not included as DSM dose-width results became dominated by extrapolated values greater than 100%, indicating that the entire rectal circumference was receiving less than or equal to the selected isodose level. This was identified as a limitation of the ellipse fitting method when seeking to analyse low dose toxicity correlations. Doses greater than 70 Gy were also excluded from toxicity analyses due to the increasing frequency of 0% DSM dose-widths, indicating that doses greater than or equal to the selected isodose level were not received by the rectal wall. Only 49/109 patients recorded a non-zero result from accumulated DSM at 74 Gy, reducing to 10/109 at 75 Gy, compared with 106/109 and 64/109 respectively from planned-DSM. It was identified that a 0% DSM result could conceal information leading to misinterpretation of data when performing AUC calculations so results at these isodose levels were not reported. Despite these restriction, the dose levels included within this study incorporate the 39–61 Gy range at which Buettner [1] determined significant correlations between lateral extent and toxicity.

**Fig. 2:**
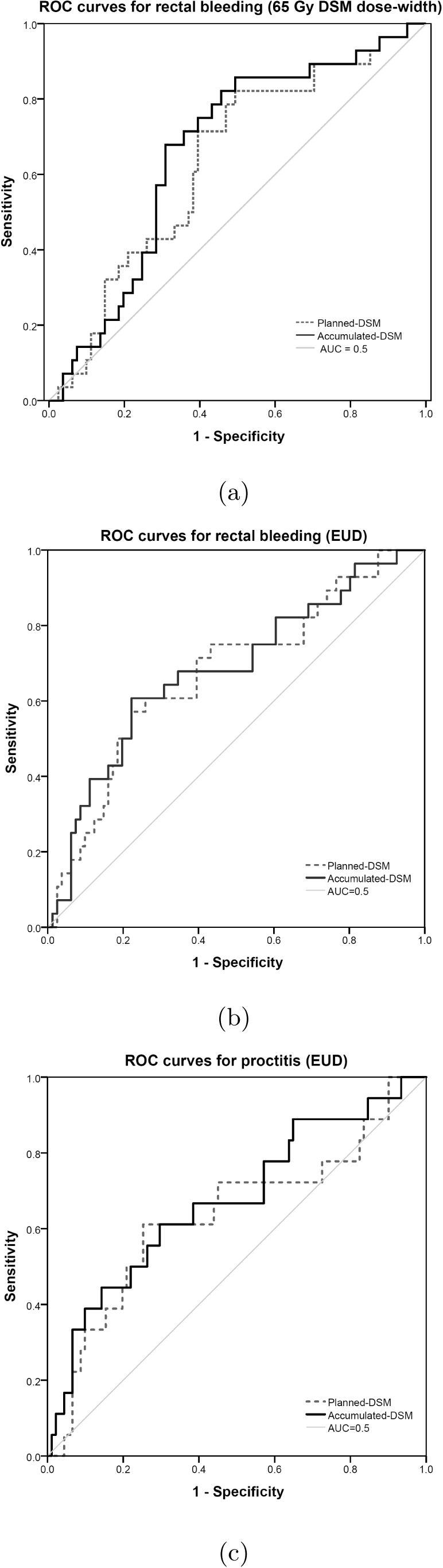
Receiver operator characteristic curves for (a) rectal bleeding with 65 Gy DSM dose-widths, (b) rectal bleeding with EUD, and (c) proctitis with EUD.

Study specific clinical reporting forms were developed for robust collection of toxicity data, and raw data were used to populate recognised systems, including: Common Terminology Criteria for Adverse Events (CTCAE) v4.03 [29], Late Effects of Normal Tissues/Subjective, Objective, Management, Analytic (LENT SOMA) scores [30]; Radiation Therapy Oncology Group (RTOG) grading system [31]; University of California, Los Angeles, Prostate Cancer Index (UCLA-PCI) questionnaire [32]. Receiver Operator Characteristic (ROC) curves (Fig. 2) were generated using SPSS® (IBM® 23.0.0.2) to evaluate the link between dosimetric parameters extracted from planned and accumulated DSMs, and the six most prevalent clinical endpoints, listed in Table 2.

**Table 2:** Clinical endpoints, scoring systems and incidence rates of the 6 most frequently reported toxicities within the patient sample (^*^Data were missing for 4 patients so sample size was reduced accordingly).

Clinical Endpoint	Scoring System	Incidence % (*n*)
Rectal Bleeding ⩾Grade 1 (Rectal Bleeding ⩾Grade 2)	CTCAE [29] (LENT SOMA [30])	25.7 (28/109)
Proctitis ⩾Grade 2	RTOG [31]/ Gulliford [35]	16.5 (18/109)
Sphincter Control ⩾Grade 1	LENT SOMA [30]	10.1 (11/109)
Rectal Pain ⩾Grade 1	CTCAE [29]/ LENT SOMA [30]	15.6 (17/109)
Bowel bother ⩾Grade 1	UCLA-PCI [32]	30.7 (32/105^*^)
Bowel bother ⩾Grade 2	UCLA-PCI [32]	11.5 (12/105^*^)

The mean area under the curve (AUC), with associated upper and lower 95% confidence intervals (CIs), was calculated for each ROC curve as a measure of the level of association between dosimetric parameter and toxicity. An ideal correlation would have an AUC of 1. Results were reported for dosimetric parameters with AUC ⩾ 0.6 and lower 95% CI ⩾ 0.5, considered statistically significant by Gulliford et al. [33].

## Results

### Rectal bleeding

**Fig. 3:**
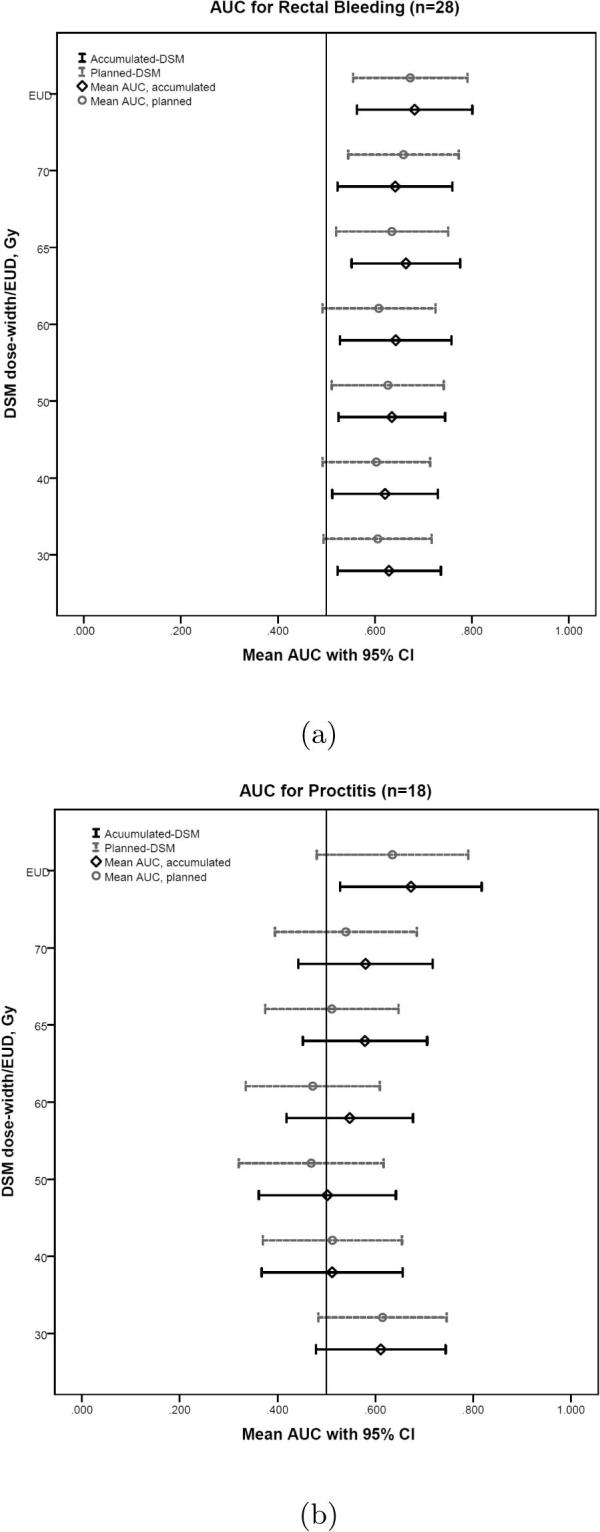
High-low plots of mean AUC and 95% confidence intervals for (a) Rectal Bleeding and (b) Proctitis, where results were considered significant if mean AUC ⩾0.6 and lower 95% CI ⩾0.5.

Twenty-eight patients reported rectal bleeding CTCAE ⩾Grade 1, which was equivalent to ⩾Grade 2 (LENT SOMA). The AUC was greater for all accumulated DSM dose-widths than planned DSM dose-widths up to 70 Gy (Table 3). At 30, 40 and 60 Gy, the lower 95% CI boundaries for the planned DSM dose-widths extended below 0.5, but remained above this threshold for the corresponding accumulated DSM dose-widths (Fig. 3a). The strongest spatial predictor of rectal bleeding was accumulated 65 Gy DSM dose-width (AUC 0.664), and the largest difference between planned and accumulated DSM dose widths was at 60 Gy (AUC difference 0.035).

**Table 3:** Mean Area Under the Curve (AUC) for planned and accumulated DSM dose-widths and EUD corresponding to rectal bleeding ⩾Grade 2 (LENT SOMA) and ⩾Grade 1 (CTCAE), *n* = 28/109. The greater AUC of each parameter has been presented in bold.

Dose Level (Gy)	Mean AUC (Planned)	Mean AUC (Accumulated)
30	0.606	**0.629**
40	0.603	**0.621**
50	0.627	**0.635**
60	0.608	**0.643**
65	0.635	**0.664**
70	**0.659**	0.642

EUD	0.673	**0.682**

Overall, for both planned and accumulated DSMs, the AUC was greater for EUD than from respective DSM dose-widths, with the strongest predictor of rectal bleeding being accumulated-EUD (AUC 0.682).

### Proctitis

The RTOG definition of proctitis considers urgency and frequency of bowel movements, as well as the presence of rectal mucous/blood. Eighteen patients reported RTOG proctitis ⩾Grade 2. Accumulated-EUD (AUC 0.673) was the only dosimetric parameter with AUC ⩾ 0.6 and lower 95% CI ⩾ 0.5 (Fig. 3b). Accumulated DSM dose-widths had greater AUC than planned DSM dose-widths at 50, 60, 65 and 70 Gy, and were equivalent at 40 Gy. At 30 Gy, the AUC of planned DSM dose-width was slightly higher than the accumulated AUC (0.004 difference).

### Remaining clinical endpoints

For the remaining clinical endpoints (LENT SOMA sphincter control ⩾Grade 1; CTCAE/LENT SOMA subjective rectal pain ⩾Grade 1; UCLA-PCI “Overall, how big a problem have your bowel habits been for you during the last 4 weeks?”, bowel bother ⩾Grade 1 and ⩾Grade 2), EUD and DSM dose-widths had little discriminatory power from planned-DSM or accumulated-DSM. No dosimetric parameter was found to have AUC ⩾ 0.6 and lower 95% CI ⩾ 0.5. Results have been included as supplementary data.

### Equivalent uniform dose

EUD produced the greatest AUCs for rectal bleeding and proctitis, indicating a stronger association than the spatial parameters investigated. In both cases, accumulated-EUD generated a higher AUC than planned-EUD. For all patients, EUD of accumulated-DSM was lower than that of planned-DSM (mean difference −2.2 Gy, standard error 0.3 Gy, range [−0.3, −7.1] Gy).

## Discussion

Radiation dose received by the rectal wall during prostate radiotherapy was calculated and accumulated using DSMs. Geometric aspects of dose distribution - information not distinguishable from DVHs – were parametrised using DSM dose-widths. EUD was calculated to compare planned and accumulated DSMs using a single metric. Extracted dosimetric parameters were evaluated against six clinical endpoints reported by patients within the VoxTox research programme. Previous dose-toxicity investigations in the literature have been limited to planned dose only. This study has demonstrated, for the first time, that delivered dose can be a stronger predictor of toxicity in the case of rectal bleeding and proctitis in prostate radiotherapy.

Toxicity rates reported in the literature have been variable. The rate of bowel toxicity ⩾Grade 2 (CTCAE), 5 year cumulative incidence, amongst VoxTox prostate patients was 17%. This falls within the bowel toxicity ⩾Grade 2 (RTOG) range of 13.7–24.9% for IMRT over the same timeframe, reported by Dearnaley et al. [9] and Wortel et al. [34], respectively. The rates of incidence indicate that toxicity remains an important clinical issue.

Many associations were found between DSM dose-widths with rectal bleeding. Accumulated DSMs generated greater AUCs than planned DSMs for 5 DSM dose-width levels up to 70 Gy. The strongest correlation between rectal bleeding and any spatial parameter was the 65 Gy DSM dose-width from accumulated dose (AUC 0.664). At 30, 40 and 60 Gy, accumulated DSM dose-widths produced AUC ⩾ 0.6 and lower 95% CI ⩾ 0.5, where corresponding planned DSM dose-widths did not. These thresholds were considered indicative of significance following the methods of Gulliford et al. [33]. The greatest difference between planned and accumulated AUCs was observed at the 60 Gy DSM dose-width. Overall, the results compared well with the findings of Buettner et al. [1] who reported the most significant correlation with rectal bleeding to be the 61 Gy lateral extent (AUC 0.66), derived from planned dose data.

Accumulated EUD was found to have the strongest correlation overall with rectal bleeding (AUC 0.682), and was the only predictor of proctitis (AUC 0.673).

For all patients, accumulated-EUD was systematically lower than planned-EUD. A contributory factor was possibly the inherent blurring of high dose regions during accumulation. Upon visual inspection of daily DSMs, the differences in size, shape and position of the high dose region due to anatomical variation was clearly visible (for example, shown in deep red in Fig. 1). During accumulation, high doses were superimposed in overlap regions, but reduced where isodose edges differed, due to averaging over the full course of radiotherapy. This affected the maximum dose of the accumulated-DSM, on which EUD calculation was heavily weighted.

The dose-blurring effect could also have been responsible for the increased frequency of 0% DSM dose-width results at high dose levels from accumulated-DSMs with respect to planned-DSMs. At 70 Gy, 4/109 patients recorded a 0% accumulated DSM dose-width (including 1 patient experiencing toxicity), whereas all corresponding planned DSM dose-widths had non-zero results. Furthermore, dose levels could not be considered independent variables, as a low 70 Gy DSM dose-width was likely to be associated with a low 65 Gy DSM dose-width, and a cooler plan overall. These issues were not accounted for within the scope of this study.

The generally lower reported values for EUD and DSM dose-widths from accumulated dose compared with planned dose should not be interpreted as delivered treatment erring on the ’safe side’ in terms of dose to rectum. Where current NTCP models are based on planned dose, the presented results suggest that the same magnitude of risk would be associated with a systematically lower delivered dose.

The findings show that the difference in dose between patients with and without rectal toxicity is greater from delivered dose than planned dose. This indicates that dosimetric parameters from accumulated-DSMs could provide new information to improve understanding of the relationship between dose and toxicity. The single parameter EUD was a superior predictor of rectal bleeding and proctitis than spatial dose quantifiers. However, DSM-dose widths produced several strong correlations with rectal bleeding, and for 5/6 dose levels, accumulated dose generated AUC values greater than planned dose.

The ability to preserve and accumulate spatial dose information throughout treatment is a novel process requiring careful consideration of data interpretation and parametrisation. Future work may involve exploring alternative methods for geometrical quantification of spatial dose distributions in order to determine stronger correlations with toxicity. Analysis of delivered dose to the rectal wall could facilitate the identification of inhomogeneous intra-organ radiosensitivities, allowing shape-based dose constraints to be derived. Spatial considerations could complement current DVH-based approaches to treatment planning.

Through novel characterisation of delivered dose, beyond the limitations of the static planned DVH, the aim is to determine those parameters strongly associated with rectal toxicity which could be incorporated into multivariate NTCP models. Emerging dose quantifiers could be integrated into planning constraints, as well as being prospectively monitored throughout treatment. Delivered dose can be accumulated in ’real-time’ and analysed with each fraction, allowing on-treatment toxicity risk assessment. Towards the end of the course of treatment, if toxicity prediction was found to be lower than planned, the decision could be made to increase the total delivered dose to the target. The potential scope for further individualisation and adaptation of treatment could ultimately reduce rates of toxicity incidence and improve clinical outcomes.

## Conclusion

Parametrisation of delivered dose to the rectal wall during prostate radiotherapy has revealed stronger correlations with rectal bleeding and proctitis than achievable from planned dose. New information from accumulated delivered dose could lead to improved dose-toxicity modelling in the future, with the aim of reducing post-treatment toxicity.

## Conflict of interest

The authors declare no conflicts of interest.
