# Supplementary material for: Delivered dose can be a better predictor of rectal toxicity than planned dose in prostate radiotherapy
Source: Radiother Oncol. 2017 Jun;123(3):466–71. doi: 10.1016/j.radonc.2017.04.008 (PMC5486775; doi:10.1016/j.radonc.2017.04.008)
Supplement: Supplementary file 1 [file mmc1.pdf]

Appendix A

Rectal Bleeding

Table 1: Mean Area Under the Curve (AUC) and 95% confidence intervals for planned and accumulated DSM dose-widths and EUD corresponding to rectal bleeding $\geq$ Grade 2 (LENT SOMA) and $\geq$ Grade 1 (CTCAE), n = 28/109. For plot, see Figure 1.

Dose Level (Gy)	Planned AUC [95% CI]	Accumulated AUC [95% CI]
30	0.606 [0.494, 0.717]	0.629 [0.523, 0.736]
40	0.603 [0.492, 0.714]	0.621 [0.512, 0.730]
50	0.627 [0.511, 0.742]	0.635 [0.525, 0.745]
60	0.608 [0.492, 0.725]	0.643 [0.528, 0.758]
65	0.635 [0.520, 0.751]	0.664 [0.552, 0.776]
70	0.659 [0.545, 0.773]	0.642 [0.523, 0.760]
EUD	0.673 [0.555, 0.791]	0.682 [0.563, 0.801]

Proctitis

Table 2: Mean Area Under the Curve (AUC) and 95% confidence intervals for planned and accumulated DSM dose-widths and EUD corresponding to Proctitis $\geq$ Grade 2 (Gulliford/RTOG), n = 18/109. For plot, see Figure 2.

Dose Level (Gy)	Planned AUC [95% CI]	Accumulated AUC [95% CI]
30	0.615 [0.484, 0.746]	0.611 [0.479, 0.744]
40	0.512 [0.370, 0.654]	0.512 [0.367, 0.656]
50	0.469 [0.321, 0.617]	0.502 [0.362, 0.642]
60	0.472 [0.335, 0.609]	0.548 [0.418, 0.677]
65	0.511 [0.375, 0.647]	0.579 [0.452, 0.706]
70	0.540 [0.395, 0.685]	0.580 [0.443, 0.717]
EUD	0.635 [0.480, 0.790]	0.673 [0.528, 0.817]

Sphincter Control

Table 3: Mean Area Under the Curve (AUC) and 95% confidence intervals for planned and accumulated DSM dose-widths and EUD corresponding to Sphincter Control ≥ Grade 1 (LENT SOMA), n = 11/109. For plot, see Figure 3.

Dose Level (Gy)	Planned AUC [95% CI]	Accumulated AUC [95% CI]
30	0.531 [0.330, 0.732]	0.533 [0.335, 0.732]
40	0.572 [0.344, 0.800]	0.582 [0.356, 0.807]
50	0.518 [0.307, 0.729]	0.525 [0.332, 0.718]
60	0.491 [0.290, 0.691]	0.558 [0.373, 0.742]
65	0.491 [0.301, 0.681]	0.557 [0.361, 0.752]
70	0.532 [0.325, 0.738]	0.619 [0.435, 0.802]
EUD	0.474 [0.282, 0.666]	0.519 [0.330, 0.707]

Rectal Pain

Table 4: Mean Area Under the Curve (AUC) and 95% confidence intervals for planned and accumulated DSM dose-widths and EUD corresponding to Rectal Pain ≥ Grade 1 (LENTSOMA/CTCAE), n = 17/109. For plot, see Figure 4.

Dose Level (Gy)	Planned AUC [95% CI]	Accumulated AUC [95% CI]
30	0.556 [0.396, 0.717]	0.543 [0.379, 0.707]
40	0.449 [0.289, 0.609]	0.403 [0.238, 0.569]
50	0.323 [0.162, 0.483]	0.297 [0.155, 0.438]
60	0.327 [0.192, 0.462]	0.386 [0.255, 0.517]
65	0.369 [0.232, 0.506]	0.437 [0.311, 0.564]
70	0.407 [0.260, 0.555]	0.545 [0.416, 0.674]
EUD	0.542 [0.400, 0.683]	0.581 [0.445, 0.718]

Bowel Problems $\geq$ Grade 1

Table 5: Mean Area Under the Curve (AUC) and 95% confidence intervals for planned and accumulated DSM dose-widths and EUD corresponding to Bowel Problems $\geq$ Grade 1 (Gulliford), n = 32/105. For plot, see Figure 5.

Dose Level (Gy)	Planned AUC [95% CI]	Accumulated AUC [95% CI]
30	0.543 [0.423, 0.663]	0.543 [0.422, 0.665]
40	0.467 [0.351, 0.583]	0.438 [0.322, 0.554]
50	0.419 [0.299, 0.538]	0.404 [0.284, 0.524]
60	0.443 [0.319, 0.566]	0.476 [0.352, 0.601]
65	0.476 [0.355, 0.596]	0.522 [0.401, 0.643]
70	0.502 [0.379, 0.624]	0.540 [0.415, 0.664]
EUD	0.515 [0.395, 0.635]	0.533 [0.413, 0.653]

Bowel Problems $\geq$ Grade 2

Table 6: Mean Area Under the Curve (AUC) and 95% confidence intervals for planned and accumulated DSM dose-widths and EUD corresponding to Bowel Problems $\geq$ Grade 2 (Gulliford), n = 12/105. For plot, see Figure 6.

Dose Level (Gy)	Planned AUC [95% CI]	Accumulated AUC [95% CI]
30	0.611 [0.445, 0.777]	0.590 [0.412, 0.767]
40	0.418 [0.281, 0.556]	0.357 [0.217, 0.496]
50	0.289 [0.143, 0.436]	0.243 [0.121, 0.365]
60	0.230 [0.108, 0.352]	0.282 [0.150, 0.415]
65	0.279 [0.148, 0.410]	0.355 [0.213, 0.496]
70	0.311 [0.158, 0.464]	0.409 [0.258, 0.561]
EUD	0.330 [0.166, 0.494]	0.352 [0.210, 0.494]

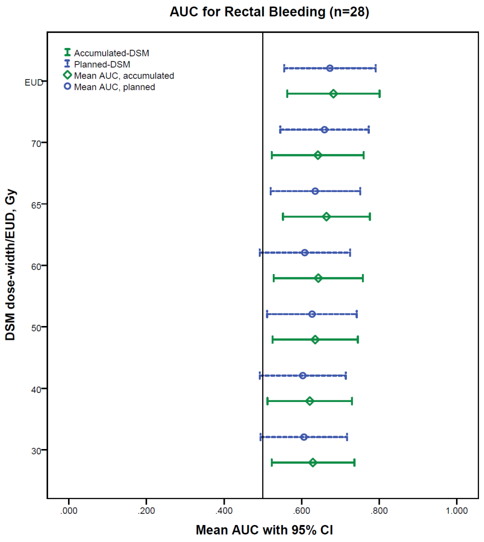

Figure 1: High-Low plot of mean AUC and 95% CI for rectal bleeding

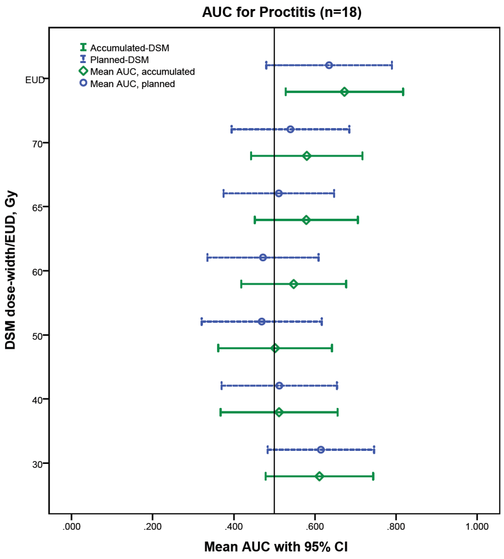

Figure 2: High-Low plot of mean AUC and 95% CI for proctitis

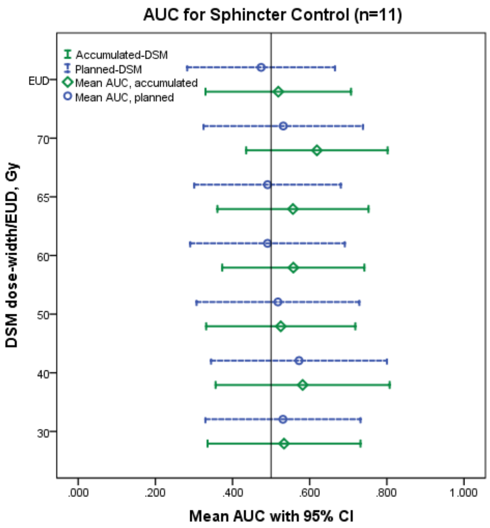

Figure 3: High-Low plot of mean AUC and 95% CI for sphincter control

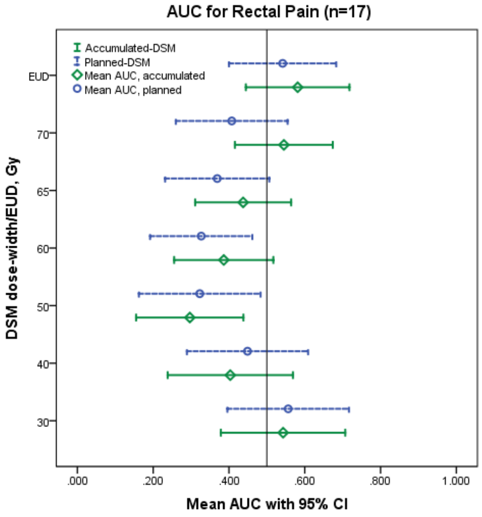

Figure 4: High-Low plot of mean AUC and 95% CI for rectal pain

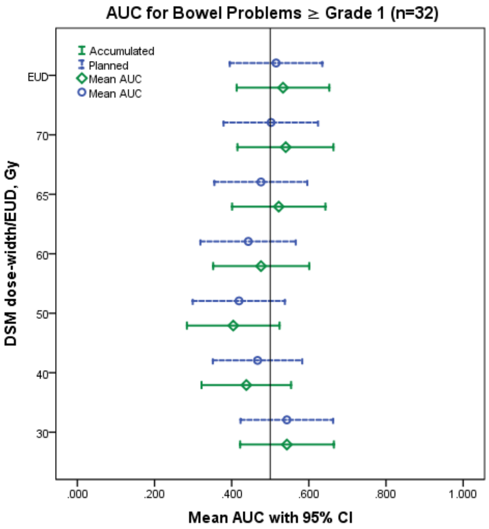

Figure 5: High-Low plot of mean AUC and 95% CI for bowel problems $\geq$ grade 1

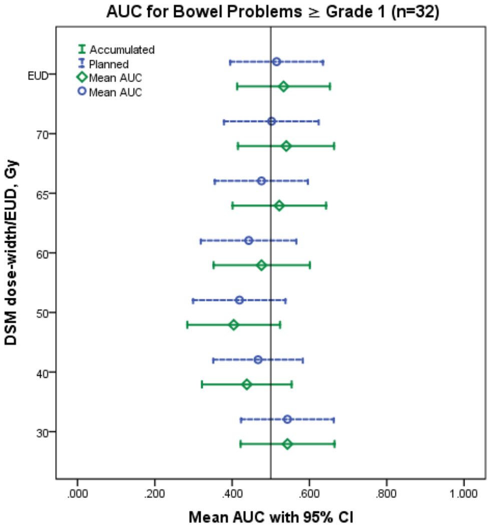

Figure 6: High-Low plot of mean AUC and 95% CI for bowel problems $\geq$ grade 2
